# The pharmacokinetic and residue depletion study of eugenol in carp (*Cyprinus carpio*)

**DOI:** 10.3389/fvets.2022.1097812

**Published:** 2023-01-25

**Authors:** Yidan Xu, Yaqin Jiao, Jian Yang, Aijuan Tan, Deyuan Ou, Xuqin Song, Shiming Lv

**Affiliations:** ^1^Laboratory of Animal Genetics, Breeding and Reproduction in the Plateau Mountainous Region, Ministry of Education, Guizhou University, Guiyang, Guizhou, China; ^2^College of Life Science, Guizhou University, Guiyang, Guizhou, China

**Keywords:** high performance liquid chromatography, eugenol, carp, pharmacokinetic, depletion

## Abstract

**Introduction:**

The pharmacokinetic profile and residue depletion of eugenol in carp (*Cyprinus carpio*) tissues and plasma were performed by a convenient and reliable high-performance liquid chromatography (HPLC) method.

**Methods:**

The eugenol in carp tissues and plasma was extracted with a mixed solution of acetonitrile and methanol. N-hexane was used to remove lipid impurities. The method was successfully applied to the pharmacokinetic and residue elimination of eugenol in carp after the carp was administered a medicated bath.

**Results:**

The average recoveries of eugenol in tissues and plasma fortified with four concentration levels were 69.0–106.6% and 80.0–86.7%, respectively. The relative standard deviations were < 8.9%. The limit of detection (LOD) was 0.01 μg/g in tissue and 0.008 μg/ml in plasma, respectively. The pharmacokinetic parameter of C_max_ for eugenol in plasma at the concentrations of 20, 35, and 75 mg/L were 10.86, 17.21, and 37.32 mg/L, respectively. The t_1/2_ values were 3.68, 4.22, and 9.31 h. After the investigation of the anesthetic effect, 35 mg/L of eugenol was the optimal concentration for anesthesia. The highest accumulation concentration of eugenol in carp is in the liver and the lowest is in the muscle. In addition, the eugenol in tissue was eliminated rapidly and at a lower level than the LOD at 48 h. According to the residue elimination, the withdrawal time of eugenol was suggested at 5.2 days.

**Discussion:**

These results indicate that the developed method had good linearity and accuracy, and is sensitive enough for the monitoring of eugenol residue in carp. The half-life of eugenol decreased with the increase in drug concentration and the eugenol was eliminated rapidly in carp tissues. 35 mg/L eugenol was recommended as an anesthetic in carp due to its favorable anesthetic effect and no mortality. This study will contribute to the establishment of MRL regulation and setting a withdrawal period.

## Introduction

Eugenol is a phytogenic bioactive component that can be extracted from multiple herbal plants such as clove, basil, and laurel. With the extensive botanical origin, low cost, and simple extraction of eugenol, it has been practiced in numerous aspects such as anti-inflammatory, antibacterial, abirritation, and anesthetization (1). In dentistry, eugenol is commonly used as a filling tooth material against pain or bacterial infection. Since eugenol was proven to have a good anesthetic effect on fish in the 1980s, it has been widely used in the fishing and transportation of fish, shrimp, and many other aquatic animals (2–4). Eugenol could effectively reduce the injury and mortality of aquatic animals by making them enter temporary dormancy and slowing down their physiological activity during transportation. A study reported by Zahran et al. (5) confirmed that eugenol had an excellent anesthetic effect and no significant influence on liver function enzymes and superoxide dismutase in Nile tilapia (5). Viegas et al. (6) found that 50 mg/L of eugenol had an effective anesthetic effect on neotropical fish and no gill damage was observed, which benefits the good health and fast recovery of the fish.

Despite the considerable success of eugenol as an anesthetic in the fishery industry, the safety of eugenol and its residual risks remain controversial. The National Toxicology Program of America conducted a 103-week research on the toxicity of eugenol (7). The results showed that eugenol did not cause cancer or chromosome mutations in rats during the experiment. However, the European Food Safety Authority suggested that more studies are needed to evaluate the toxicity of eugenol because there is no more evidence that eugenol is carcinogenic after a 2-year chronic toxicity study (8). In addition, the International Agency for Research on Cancer regarded eugenol as a possible carcinogen in Group 3 because of its ambiguous carcinogenicity to humans (9). Therefore, the regulation on whether eugenol could be authorized as an anesthetic in fish varies by country. The Food and Drug Administration allows eugenol as a food additive, while it is not approved for use as an anesthetic in fish (10). In Japan, the application of eugenol to fish is legal, and the maximum residue limit (MRL) in fish is 0.05 mg/kg (11). The China and European Union have not issued any regulation on whether eugenol could be used as an anesthetic and its MRL in fish. With the rapid development of the fishery, eugenol is more and more widely used as an anesthetic. It is reported that eugenol was detected with the highest concentration of 30,690 μg/kg in fish in wholesale markets in China, and the detection rate of eugenol residue was higher than 10% (12). Considering the high applied concentration of eugenol, its toxicity, pharmacokinetic profile, and residue deserve high attention.

Currently, the analysis of eugenol residue in animal tissue includes high-performance liquid chromatography (HPLC), liquid chromatography tandem mass spectrometry, and gas chromatography mass spectrometry (13–15). The HPLC has the advantages of rapid detection, high popularity, and few matrix interferences, becoming a major method in the pharmacokinetic study. Carp (*Cyprinus carpio*) is one of the main species of edible freshwater fish in China. It is popular with consumers for its delicious meat, low cost, and rich nutrition. Because carp are prone to injury during transportation, fishing, and sale, eugenol is commonly used as an anesthetic (16–18). However, the use of eugenol as an anesthetic in carp may lead to drug residue, which could pose a threat to humans. More importantly, little data about the pharmacokinetics of eugenol and its residue elimination in carp could be available. Therefore, a rapid, simple, and sensitive method for the determination of eugenol in carp plasma and tissue was established and applied to the study of pharmacokinetics and depletion, which will provide basic data for the residual risk assessment of eugenol used as an anesthetic in carp.

## Materials and methods

### Reagents and materials

Eugenol with a purity higher than 98% was purchased from J&K Scientific (Beijing, China). HPLC grade solvents of methanol (MeOH), acetonitrile (ACN), and ethanol (EtOH) were bought from Bioengineering Co., Ltd (Shanghai, China). Heparin sodium was bought from Beijing Solarbio Science and Technology Co., Ltd (Beijing, China). N-hexane was purchased from Shandong Xiya Chemical Technology Co., Ltd (Shandong, China).

A total of 10 mg of eugenol was dissolved in 10 ml of MeOH to prepare a stock solution (1 mg/ml), which could be stored at −20°C for up to 3 months. A working standard solution was prepared by diluting the stock solution daily.

### Animals and captivity conditions

In this study, 400 carp with an average weight of 750 ± 50 g were used. They were acquired from a private fish farm (Guiyang, China). The carp were kept in several 500 L fiberglass tanks with a continuous flow of tap water filtered by activated carbon. An oxygen pump provides continuous aeration, and the dissolved oxygen should be more than 6.0 mg/L. The water temperature was maintained at 24 ± 1°C. The ammonia-nitrogen content should keep < 0.2 mg/L. The water pH was maintained at 7.20 ± 0.25. The carp were acclimatized for 3 days prior to experimentation. The experimental protocol was approved by the Subcommittee of Experimental Animal Ethics of Guizhou University (No: EAE-G2u-2020-P027, 3 December 2020) prior to animal use.

### Sample preparation

Homogeneous tissue of 2 g was weighed into a 15-ml polypropylene centrifuge tube. An appropriate working standard solution of eugenol was spiked into tissues to prepare quality control samples. Samples were allowed to incubate for 30 min to ensure the permeation of analytes into the tissue. The eugenol in tissue was ultrasonically extracted with 5 ml of an equal proportion of ACN and MeOH for 10 min. The extract was centrifuged at 10,000 rpm for 10 min at 4°C. The supernatant was transferred to a 50 ml polypropylene centrifuge tube, and the residue was extracted again. After centrifugation, the supernatants were combined and 6 ml of n-hexane was used to remove co-extracted impurities. After centrifugation at 8,000 rpm for 6 min, the n-hexane was discarded, and the extract solution was evaporated to dryness under nitrogen at 45°C. The residue was reconstituted with 0.5 ml of the MeOH-water solution (1:1, v:v) for the HPLC analysis.

A total of 0.5 ml of plasma was sucked into a 2-ml centrifuge tube, and 1 ml of the equal proportion of ACN and MeOH was used to extract equal parts by vortexing for 2 min. After centrifugation, grease removal with 2 ml of n-hexane, evaporation, and residue were dissolved in 0.2 ml of reconstituted solution.

### HPLC analysis

The HPLC system included an Agilent Technologies 1260 series chromatograph (Agilent Technologies, Santa Clara, CA) equipped with a diode array detector (DAD). The separation was achieved using an Agilent SB-C_18_ (4.6 × 150 mm i.d., 5 μm). The mobile phase was composed of ACN, MeOH, and water (31:31:38, v:v:v), and the analyte was eluted through isocratic elution with a constant flow rate of 1 ml/min. The ultraviolet wavelength was set to 280 nm, and the injection volume was 20 μl.

### Method validation

According to the European Commission Decision 2002/657/EC, method validation including selectivity, linearity, accuracy and precision, limit of detection (LOD), and limit of quantification (LOQ), was performed.

A total of 100 blank samples (plasma, muscle, liver, kidney, and brain, 20 each) were analyzed to estimate the selectivity of the developed method under the optimal pretreatment conditions. Endogenous substances cannot infect the target analyte.

The linearity of the developed method was performed by analyzing six concentration levels of eugenol (0.1, 0.2, 1, 5, 10, and 20 μg/ml). The standard curve originated from using the peak area of eugenol vs. the corresponding concentration in the solution. The coefficient of correlation (*r*^2^) must be higher than 0.99.

Blank carp tissue samples (muscle, liver, kidney, and brain) spiked with four concentration levels of LOQ (1, 5, and 10 μg/g) and blank carp plasma samples spiked with four concentration levels of LOQ (0.1, 1, and 10 μg/ml) were used to evaluate accuracy (recovery) and precision. The precision is expressed by the relative standard deviation (RSD). The intra-day and inter-day RSDs were assessed by analyzing quality control samples (six replicates for each level) at the same concentration on the same day and on three different days, respectively.

The LOD and LOQ were calculated according to the signal-to-noise ratios of 3:1 and 10:1, respectively.

### Application

The developed method was applied to the pharmacokinetic and residue elimination of eugenol in carp.

#### Pharmacokinetic study

After a 3-day acclimation, the carp fasted for 24 h before the experiment. The carp were randomly divided into 27 treatment groups and a blank group, with 10 carp in each group. The carp were then immersed individually in a solution of 75 mg/L of eugenol for 15 min, and the medicine bath administration was performed according to the literature (19, 20). The appropriate eugenol was first diluted in ethanol (1:10, v/v) and then added to water to keep the immersion concentration of eugenol at 20, 35, and 75 mg/L. The carp were immersed in three concentration levels of eugenol (20, 35, and 75 mg/L) for 15 min. Then, each group of carp was rinsed thoroughly with water and placed in another fiberglass tank. Blood samples were collected from the tail vein or heart at 0.25, 0.5, 1, 2, 4, 6, 8, 24, and 48 h after administration (10 carps for each time point). Blood samples were collected in centrifuge tubes with heparin sodium. After centrifugation at 8,000 rpm for 10 min, plasma was collected and frozen at −20°C until analysis.

#### Residue elimination

The carp were randomly divided into 10 treatment groups and a blank group with 10 carp for each group. A single dose of eugenol (35 mg/L) was administered to the carp *via* medicated bath. Carp tissues including muscle, liver, kidney, and brain were collected at 10 min, 0.25, 0.5, 1, 2, 4, 8, 16, 24, and 48 h and were frozen at −20°C until analysis.

### Data analysis

The Excel 2010 version was used to calculate the recovery and standard deviation of eugenol in carp. Concentrations of eugenol in plasma were reported as the mean ± SD at each time point. Depletion profiles of eugenol in plasma were estimated by a non-compartmental analysis using WinNonlin 8.1.0.3530. The elimination rate constant in plasma (K) was calculated, and the elimination half-life (t_1/2_) was calculated according to $\frac{0.693}{K}$. The area under the concentration vs. time curve from time zero extrapolated to infinity (AUC_0−∞_) was calculated using the linear trapezoidal method. The peak concentration (C_max_) and time to reach the maximum concentration (t_max)_ were directly obtained from the concentration vs. time data. The concentrations of eugenol in carp plasma at different time points were compared by an independent sample *t*-test with IBM SPSS Statistics 26 software. Statistics analysis was performed with SPSS version 26.0, and *P* < 0.05 was considered statistically significant. The withdrawal period of eugenol was calculated by the off-drug period calculation software WT1.4.

## Results

### Method validation of the developed HPLC method

The process of sample preparation was slightly optimized based on the method in the reported literature (21). In this study, MeOH, ACN, ammonia in ACN (pH = 9.5), and MeOH-ACN (1:1, v:v) were used to extract eugenol from carp muscle. As shown in Supplementary Figure 1, MeOH and MeOH-ACN (1:1, v:v) provided satisfactory recovery for eugenol (higher than 90%). The recovery of eugenol extracted by MeOH-ACN (1:1, v:v) was better. In addition, the reconstruction solution of MeOH-water (1:1, v:v) could improve the stability of the baseline during HPLC analysis.

**Figure 1:**
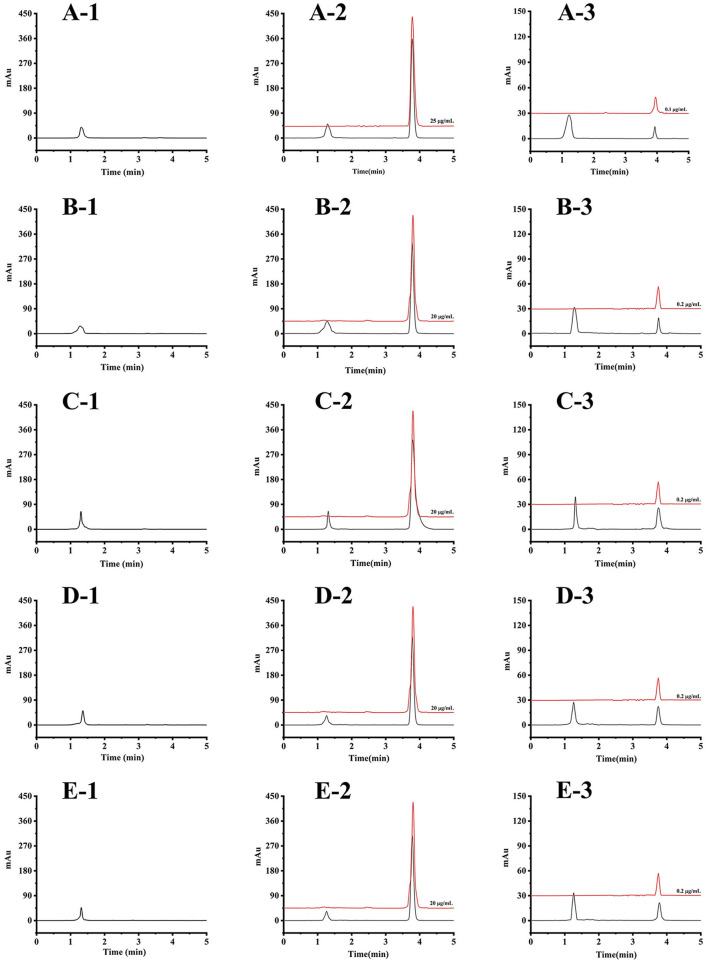
The HPLC chromatograms of blank carp matrices spiked matrices (black line) and the corresponding standard solution (red line): **(A-1)** blank plasma, **(A-2)** plasma spiked at 10 μg/ml and the standard solution of 25 μg/ml, **(A-3)** plasma spiked at LOQ concentration and the standard solution of 0.1 μg/ml; **(B-1)** blank muscle, **(B-2)** muscle spiked at 5 μg/g and the standard solution of 20 μg/ml, **(B-3)** muscle spiked at LOQ concentration and the standard solution of 0.2 μg/ml; **(C-1)** blank brain, **(C-2)** brain spiked at 5 μg/g and the standard solution of 20 μg/ml, **(C-3)** brain spiked at LOQ concentration and the standard solution of 0.2 μg/ml; **(D-1)** blank liver, **(D-2)** liver spiked at 5 μg/g and the standard solution of 20 μg/ml, **(D-3)** liver spiked at LOQ concentration and the standard solution of 0.2 μg/ml; **(E-1)** blank kidney, **(E-2)** kidney spiked at 5 μg/g and the standard solution of 20 μg/ml, **(E-3)** kidney spiked at LOQ concentration and the standard solution of 0.2 μg/ml.

The result of the selectivity test showed that no interfering peak near the retention time of the target analyte was observed, suggesting the high selectivity of the developed method. The typical chromatograms of blank carp plasma and tissue matrices and the corresponding spiked sample matrices are given in Figure 1.

The calibration curve for eugenol was *y* = 8.0371*x*+0.3918 with a linear regression coefficient (*r*^2^) of 0.9995. The calibration curve exhibited good linearity within the experimental concentration ranges.

The results for accuracy and precision are shown in Table 1. The recoveries of eugenol ranged from 69.0% (muscle) to 106.6% (kidney). The intra-day and inter-day RSDs were lower than 8.9%.

**Table 1:** Recovery and precision of eugenol in carp tissues and plasma (*n* = 6)^*a*^.

**Matrix**	**LOD (μg/g)**	**LOQ (μg/g)**	**Intra-day recovery (RSD), %**				**Inter-day recovery (RSD), %**			

			**LOQ (**μ**g/g)**	**1** μ**g/g**	**5** μ**g/g**	**10** μ**g/g**	**LOQ (**μ**g/g)**	**1** μ**g/g**	**5** μ**g/g**	**10** μ**g/g**
Liver	0.01	0.05	76.2 (8.3)	82.8 (2.0)	99.3 (2.0)	104.5 (3.1)	77.6 (7.7)	83.6 (5.0)	97.5 (2.6)	105.1 (3.6)
Muscle	0.01	0.05	69.0 (8.9)	72.4 (4.1)	92.3 (1.7)	94.7 (2.4)	73.4 (8.7)	73.0 (6.4)	95.1 (3.4)	96.3 (3.1)
Brain	0.01	0.05	91.0 (4.9)	99.4 (4.9)	96.2 (3.8)	100.4 (2.8)	91.7 (4.9)	99.8 (2.5)	97.4 (2.8)	100.0 (4.8)
Kidney	0.01	0.05	72.4 (8.9)	82.2 (5.4)	100.6 (2.7)	106.6 (4.2)	80.8 (7.4)	77.4 (4.6)	100.1 (2.5)	106.4 (5.5)
	**LOD (**μ**g/mL)**	**LOQ (**μ**g/mL)**	**LOQ (**μ**g/mL)**	**0.1** μ**g/mL**	**1** μ**g/mL**	**10** μ**g/mL**	**LOQ (**μ**g/mL)**	**0.1** μ**g/mL**	**1** μ**g/mL**	**10** μ**g/mL**
Plasma	0.008	0.04	80.2 (7.9)	86.7 (7.5)	80.3 (3.8)	80.0 (1.1)	82.5 (8.6)	87.3 (5.0)	81.2 (7.9)	79.9 (4.8)

^a^LOD, limit of detection; LOQ, limit of quantification; RSD, relative standard deviation.

The LOD and LOQ are given in Table 1. The LOD and LOQ of eugenol in carp muscle, liver, kidney, and brain samples were 0.01 and 0.05 μg/g, respectively. The LOD and LOQ in carp plasma were 0.008 and 0.04 μg/ml.

### Pharmacokinetic study of eugenol in carp plasma

**Figure 2:**
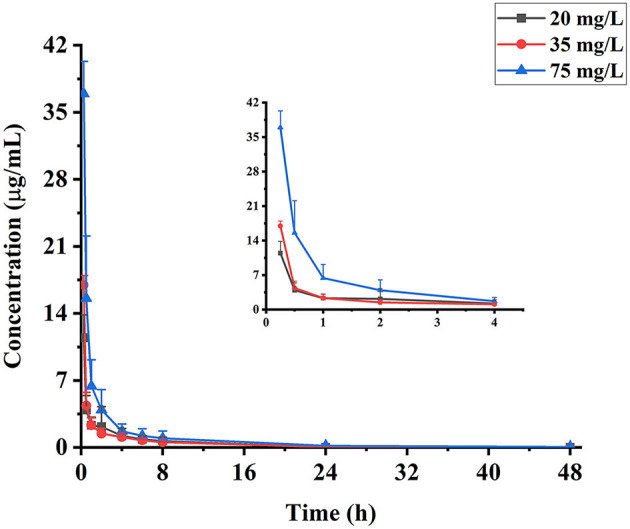
Concentration-time curves of eugenol in carp plasma collected at 0.25, 0.5, 1, 2, 4, 6, 8, 24, and 48 h (*n* = 10), following medicated bath administration of 20, 35, and 75 mg/L of eugenol.

To evaluate the pharmacokinetic profiles of eugenol in carp, plasma samples were collected at different time points after medicated bath administration of 20, 35, and 75 mg/L. The pharmacokinetic curves of eugenol in carp plasma are exhibited in Figure 2. Eugenol in carp plasma decreased rapidly at three medicated concentration levels. More than 50% of the total eugenol in plasma was eliminated within 0.5 h, and the concentration of eugenol dropped below 6.5 μg/ml after 1 h. After 48-h dosing, the residue of eugenol was few, and it could only be detected under the dose of 75 mg/L.

The depletion of eugenol in carp plasma can be described by a non-compartment model, and the pharmacokinetic parameters are presented in Table 2. Eugenol can be absorbed by carp in a short time. The depuration of eugenol in carp plasma was more suitable to be described by a non-compartment model. Under the medicated concentrations of 20, 35, and 75 mg/L, the t_1/2_ values of eugenol in carp plasma were 3.68, 4.22, and 9.31 h, which showed significant differences (*P* < 0.05). The C_max_ of eugenol in plasma is 10.86, 17.21, and 37.32 mg/L, respectively. The values of AUC_0−*t*_ were 14.15, 18.91, and 44.56 mg/L·h, respectively. The C_max_ and AUC_0−*t*_ of the eugenol increased with the administration concentration, indicating that the eugenol concentration in plasma was dose-dependent. In addition, the MRT showed the same trend with the increase in administration concentration, suggesting that the higher the dose, the longer the retention time was.

**Table 2:** Pharmacokinetic parameters of eugenol in the carp plasma (*n* = 10)^*a*^.

**Parameters**	**Unit**	**Concentrations of medicated bath**		

		**20 mg/L**	**35 mg/L**	**75 mg/L**
K	1/h	0.19 ± 0.01	0.17 ± 0.01	0.08 ± 0.01
t_1/2_	h	3.68 ± 0.1^c^	4.22 ± 0.11^b^	9.31 ± 1.03^a^
t_max_	h	0.25	0.25	0.25
C_max_	mg/L	10.86 ± 0.44^c^	17.21 ± 0.45^b^	37.32 ± 2.96^a^
AUC_0 − t_	mg/L·h	14.15 ± 0.11	18.91 ± 0.54	44.56 ± 2.49
AUC_0−∞_	mg/L·h	17.46 ± 0.07	19.16 ± 0.54	45.22 ± 2.76
MRT	h	2.50 ± 0.02^c^	3.90 ± 0.02^b^	5.29 ± 0.56^a^

^a^K, elimination rate constant; t_1/2_, half-life of elimination; t_max_, time to reach peak concentration; C_max_, peak drug concentration; AUC (0-t), area under the blood concentration-time curve (0-t); AUC (0–∞), area under the blood concentration-time curve (0–∞); MRT, mean retention time. Different lowercase letters in the figure represented significant differences (P < 0.05).

### The anesthetic effect of eugenol applied in carp

Before evaluating the residue elimination of eugenol in carp, an anesthetic effect experiment was carried out to obtain the best application drug concentration. The carp were anesthetized with eugenol at different concentrations of 20, 30, 35, and 40 mg/L in the medicated bath. The behaviors of carp at different anesthesia stages were conducted according to the reference (22). As shown in Table 3, the induction time of anesthesia for carp decreases with an increase in eugenol concentration, while the recovery time of carp is longer. The carp at the medicated bath concentration of 20 mg/L eugenols was unable to enter anesthesia stage A4 and returned to normal quickly with a recovery time of 0.5 min. Furthermore, the 30 mg/L of eugenol inducted a relatively long time for the carp to enter anesthesia stage A4 (more than 10 min). Thus, the 20 mg/L and 30 mg/L of eugenol were not suitable as the anesthetic concentration for carp. The shortest induction time (0.1 min for anesthesia stage A1) was observed at the highest concentration of 40 mg/L, while the recovery time for carp exceeded 5 min, which could increase the likelihood of death during long-time transportation. Ultimately, the optimal concentration of eugenol for the medicated bath was 35 mg/L, and under this concentration, an obvious anesthetic response (1 min for anesthesia) and excellent recovery (3.7 min) of the carp were observed.

**Table 3:** Induction and recovery time in carp at different eugenol concentrations^*a*^.

**Concentration (mg/L)**	**Induction time/s**				**Recovery time/s**		

	**A1** ^a^	**A2** ^b^	**A3** ^c^	**A4** ^d^	**W1** ^e^	**W2** ^f^	**W3** ^g^
20	135.63 ± 29.93	311.75 ± 63.66	523.25 ± 91.39	-	30.63 ± 14.50	117.13 ± 41.60	152.86 ± 51.55
30	126.00 ± 24.01	210.50 ± 38.18	434.00 ± 95.59	694.38 ± 76.13	65.90 ± 15.01	147.88 ± 30.65	197.88 ± 63.76
35	63.33 ± 23.45	63.33 ± 23.45	214.44 ± 22.42	346.88 ± 61.90	68.88 ± 14.55	159.87 ± 21.26	224.30 ± 88.08
40	10.40 ± 3.31	83.13 ± 29.39	171.88 ± 27.51	311.40 ± 94.48	88.50 ± 15.93	166.38 ± 34.51	345.00 ± 26.65

^a^Carp behavior at anesthesia period of A1: No response to loose and mild contact, but the response to strong external stimuli, increased gill cover rate. ^b^Carp behavior at anesthesia period of A2: Swim imbalance, the operculum rate is normal and responds to external stimuli. ^c^Carp behavior at anesthesia period of A3: Complete loss of balance, side lying, no response to external stimuli, gill cover rate decreased slightly. ^d^Carp behavior at anesthesia period of A4: In deep anesthesia, the frequency of the gill lids is irregular and stops from time to time. ^e^Carp behavior at convalescence of W1: Gill covers slowly open and close, stimuli are unresponsive. ^f^Carp behavior at convalescence of W2: Recovery swims slowly, and the fish body's righting reflex recovers. ^g^Carp behavior at convalescence of W3: Swim freely and all behavior returns to normal.

### Residue elimination of eugenol in carp tissues

**Figure 3:**
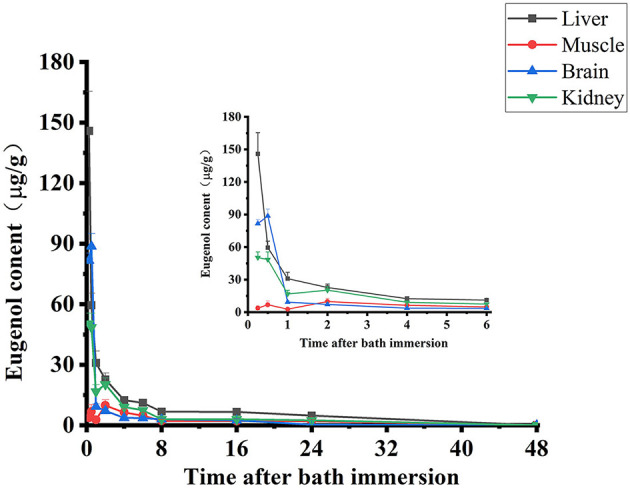
Concentration-time curves of eugenol in carp muscle, liver, kidney, and brain following a 35 mg/L eugenol of immersion bath (*n* = 10).

The depletion profiles of eugenol in carp muscle, liver, kidney, and brain are given in Figure 3. After the absorption, the concentration of eugenol reached its highest in the liver and kidney at 0.25 h, while the maximum concentration of eugenol in muscle and brain was at 1 h. During the experimental period, the residue of eugenol in the liver maintained the highest concentration among these tissues, and the highest was 145.8 μg/g at 0.25 h. Eugenol was eliminated rapidly in carp tissues, and more than 50% of the total eugenol in the liver, kidney, and brain was depleted within 1 h. After 24-h administration, only a few eugenols could be detected in four tissues, and eugenol was lower than LOD in carp tissues at 48 h. The withdrawal time was calculated based on the residue elimination data in carp muscle at five time points (23). As shown in Supplementary Figure 2, under the MRL of 0.05 mg/kg eugenol in fish established by Japan, the withdrawal time of eugenol in carp was 5.2 days.

## Discussion

Eugenol can be easily dissolved by organic solvents such as MeOH and ACN. Therefore, many reported methods for the extraction of eugenol in animal tissues and plasma used ACN (24) and ammoniated ACN (25). In this study, we compared the effects of MeOH, ACN, ammoniated ACN, and MeOH-ACN (1:1, v:v) on the recovery of eugenol in carp muscle. The MeOH-ACN (1:1, v:v) provided a higher extraction efficiency of eugenol. The MeOH could improve the liberation of eugenol from carp tissue. In addition, the participation of ACN in the extraction solution contributes to the precipitation of protein, which will show better purification. In addition, a clean-up step is necessary to improve the selectivity of the analysis. The previous studies employed solid-phase extraction (26), QuEChERS (13), and dispersive solid-phase extraction (15). Although these methods achieved the purification effect for eugenol, the handling time is long and the consumption of solvent is high. Many articles have reported a simple purification protocol for eugenol in animal tissues with the use of n-hexane. N-hexane is a non-polar solvent that could remove fat efficiently. In addition, n-hexane has low toxicity and a simple operation, thus, it is widely used in the purification of drug residues in animal-derived food residues. In this study, the selectivity of the developed method demonstrated that the extraction procedure and n-hexane defatting protocol were practicable to remove endogenous impurities. The linearity was good, and the recovery was high. Furthermore, the developed method is sensitive enough to detect eugenol, which can meet the requirements of residual analysis in carp tissues and the pharmacokinetic study of eugenol.

The eugenol was eliminated rapidly in carp after medicated bath administration. In this study, eugenol had a short elimination half-life in carp plasma (3.68, 4.22, and 9.31 h). In addition, the half-life of eugenol decreased with the increase in dosage, maybe due to the saturation of metabolic enzymes at a high dose. In practice, the half-life of eugenol varies from animal species. The half-life of eugenol at a dose of 75 mg/L in rainbow trout was 12.14 h (20), which is longer than the result at the same administration concentration in this study (2.43 h). It is reported that the pharmacokinetic profile of eugenol in Pacific white shrimp was described by a first-order kinetic mode, and the half-life was 11 h in muscle after a high eugenol concentration immersion bath (300 mg/L) (27). Hou et al. (28) investigated the pharmacokinetic study of eight volatile constituents extracted from *Artemisia argyi Folium*. The results indicated that the half-life of eugenol in rat plasma after oral administration of 125 mg/kg was 0.26 h, and its elimination rate was the fastest. Therefore, the results of the pharmacokinetic profiles in this study are similar to the findings of other literature.

Generally, eugenol should be medicated at a certain concentration to ensure its safety and reliable anesthesia for fish. The anesthetic mechanism of eugenol was proven to be associated with the GABA receptor, which is the main inhibitory neurotransmitter and is responsible for CNS depression and anesthesia (29). Although a high concentration of eugenol shortens the duration of medicated bath and improves the anesthetic effect, more deaths will occur. Accordingly, an appropriate concentration of anesthetic is necessary for fish to keep sedation and avoid long-term deep anesthesia during transportation. An ideal anesthetic should induce anesthesia within 3 min and the recovery time should be < 5 min (30). Morteza et al. (17) reported that eugenol anesthetized the carp within 600–90 s (the recovery time of 190–380 s) at concentrations of 25–150 mg/L and the long-term exposure to eugenol at high concentrations could cause oxidative stress injury and tissue damage in carp. Bowker et al. (31) assessed the effectiveness of eugenol (treated at 3 mg/L) to lightly sedate three kinds of Salmonids for 5 h in static conditions. The results showed that 3 mg/L eugenol was effective for lightly sedating juvenile salmonids for up to 5 h, which is suitable for sorting or loading fish onto a distribution truck. In addition, 53.0 mg/L eugenols were confirmed as a good anesthetic for hybrid catfish to reduce mortality and promote an increased immune system (32). In our study, 35 mg/L of eugenol for a period of 15-min immersion baths showed a favorable anesthetic effect in carp without mortality. The anesthesia and recovery times were about 1.0 min and 3.7 min, respectively.

A total of 35 mg/L of eugenol was deemed to be the appropriate anesthetic dose for carp because this dose produced sufficient deep sedation and good recovery. The results of residue elimination showed that the concentration of eugenol in the liver was higher than in other tissues at any time point, suggesting that eugenol is mainly metabolized by the liver, which agrees with the findings in the report (33). The depletion of eugenol depends on the species type and exposure time. Meinertz et al. found that the depletion of 10 mg/L 14 C-eugenol residues in Rainbow trout tissue was rapid (t_1/2_ = 26.25 min), indicating that eugenol can be safely and effectively used to sedate fish (34). Another study evaluated the effects of concentrations and duration of eugenol on the residue concentrations in rainbow trout, and the results exhibited that fish exposed to 10 mg/L eugenols for 240-min durations had the highest residue in the filet tissue (62 μg/g) (24). In addition, water temperature also plays an important role in the depletion of eugenol. According to the report (35), the t_1/2_ of eugenol in sea bass at a water temperature of 20°C was 0.29 h, while the t_1/2_ was 4.5 h at 13°C, indicating that we can increase the temperature to accelerate the depletion of eugenol in fish and reduce residue exposure. However, the withdrawal period is related to the water temperature and the type of drug, thus, it may not change much. A report described that although florfenicol was eliminated more rapidly at a higher temperature, temperature effects may not be sufficient to change the withdrawal periods of florfenicol (36). In this study, the concentrations of eugenol were the highest at 0.25 h in the liver and kidney, at 0.5 h in the brain, and 1 h in muscle, manifesting the slow accumulation of eugenol in muscle. However, eugenol was eliminated rapidly in four tissues. Especially in the liver and brain, the residue of eugenol dropped sharply at 1 h. The eugenol concentrations were lower than LOD in carp tissues at 48 h. According to the result of off-drug period calculation software, the withdrawal time of eugenol in carp was suggested 5.2 days. Nevertheless, due to the absence of accurate residue data after treatment in an eugenol bath for long-time transportation, additional studies are necessary to verify the security and residue of eugenol. The crowding environment and actual stress of transportation should be considered when establishing the MRL of eugenol in carp.

## Data availability statement

The original contributions presented in the study are included in the article/Supplementary material, further inquiries can be directed to the corresponding authors.

## Ethics statement

The animal study was reviewed and approved by the Guizhou University Subcommittee of Experinental Animal Ethics.

## Author contributions

Formal analysis and software: YX and YJ. Funding acquisition, writing—review, and editing: XS and SL. Investigation: AT and DO. Methodology: YX, YJ, and JY. Writing—original draft: YX, XS, and SL. All authors contributed to the article and approved the submitted version.
