# Supplementary material for: The pharmacokinetic and residue depletion study of eugenol in carp (Cyprinus carpio)
Source: Front Vet Sci. 2023 Jan 25;9:1097812. doi: 10.3389/fvets.2022.1097812 (PMC9905725; doi:10.3389/fvets.2022.1097812)
Supplement: Supplementary file 1 [file Data_Sheet_1.docx]

**Supplementary Materials**

**The pharmacokinetic and residue depletion study of** **eugenol**

**in** **carp (*****Cyprinus carpio*)**

**Yidan Xu ^1,^** **^†^, Yaqin Jiao ^1, †^, Jian Yang ^1^, Aijuan Tan ^2^, Deyuan Ou ^1^,**

**Xuqin Song ^1, *^ and Shiming Lv ^1, *^**

^1^Laboratory of Animal Genetics, Breeding and Reproduction in the Plateau Mountainous Region, Ministry of Education, Guizhou University, Guiyang 550025, Guizhou Province, China

^2^College of Life Science, Guizhou University, Guiyang 550025, Guizhou Province, China

* Correspondence:
Shiming Lv: [lvlvsm@163.com](mailto:lvlvsm@163.com)

* Co-correspondence:

Xuqin Song, [song1991yi@163.com](mailto:song1991yi@163.com)

† These authors contributed equally to this work and share the first authorship.


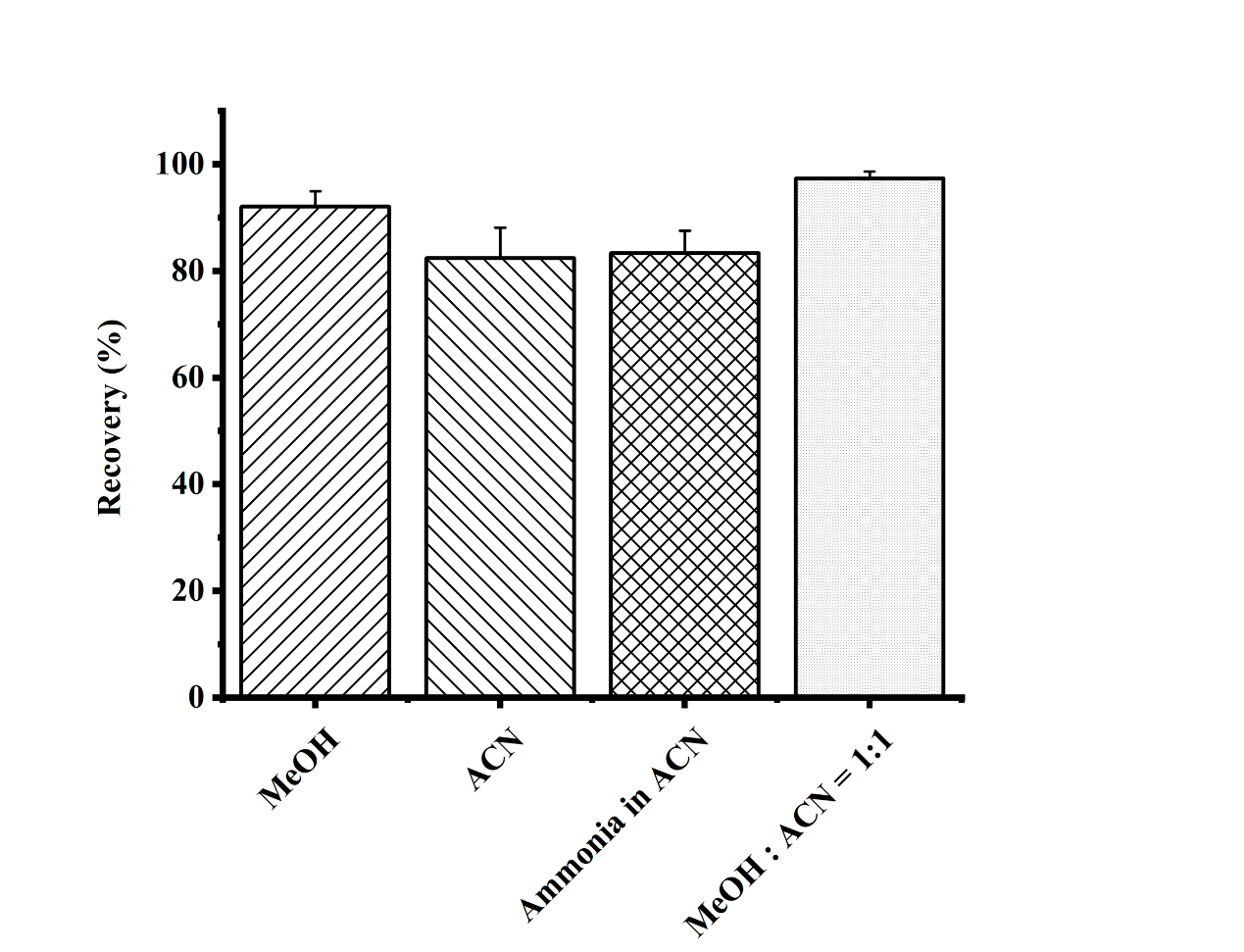


**Figure S1**  Effects of methanol (MeOH), acetonitrile (ACN), ammonia in ACN, and MeOH-ACN (1:1, v:v) on the recovery of eugenol in carp muscle


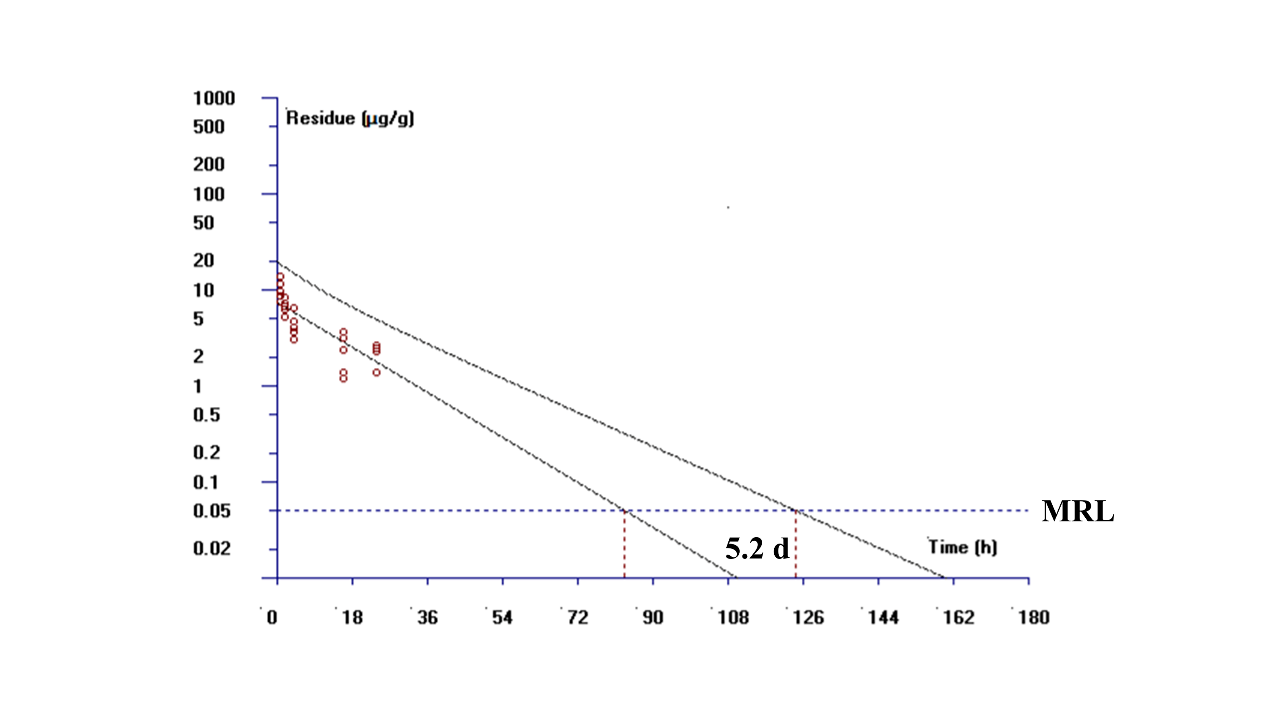


**Figure S2**  Semi-log plot of the eugenol depletion in the muscle of carp reared at 24±1°C after medicated bath. The upper regression line in the figure represents the 95th percentile.
